# Infodemia: Another Enemy for Romanian Frontline Healthcare Workers to Fight during the COVID-19 Outbreak

**DOI:** 10.3390/medicina56120679

**Published:** 2020-12-09

**Authors:** Ica Secosan, Delia Virga, Zorin Petrisor Crainiceanu, Lavinia Melania Bratu, Tiberiu Bratu

**Affiliations:** 1 Faculty of Medicine, “Victor Babes” University of Medicine and Pharmacy, 300041 Timisoara, Romania; secosan.ica@umft.ro (I.S.); zcrainiceanu@gmail.com (Z.P.C.); lavinia.melania.bratu@gmail.com (L.M.B.); office@brol.ro (T.B.); 2 Department of Psychology, West University of Timisoara, 325100 Timisoara, Romania

**Keywords:** false news, COVID-19, frontline clinicians, misinformation, stress, mental health, anxiety, insomnia

## Abstract

*Background and Objectives:* The population has been overwhelmed with false information related to the Coronavirus disease (COVID-19) crisis, spreading rapidly through social media and other channels. We aimed to investigate if frontline healthcare workers affected by infodemia show different psychological consequences than frontline clinicians who do not declare to be affected by false news related to the COVID-19 pandemic. *Materials and Methods:* One hundred twenty-six frontline healthcare workers from the Intensive Care Unit (ICU) and Emergency Departments in Romania completed a survey to assess stress, depression, anxiety, and sleep disorders, between March and April 2020. We split the sample of frontline healthcare workers into two groups based on the self-evaluated criteria: if they were or were not affected by infodemia in their activity. *Results:* Considering limitations such as the cross-sectional design, the lack of causality relationship, and the sample size, the results show that, the frontline medical workers who declared to be affected by false news were significantly more stressed, felt more anxiety, and suffered more from insomnia than healthcare workers who are not affected by false information related to pandemic time. *Conclusions:* The infodemia has significant psychological consequences such as stress, anxiety, and insomnia on already overwhelmed doctors and nurses in the outbreak of the COVID-19 crisis. These findings suggest that medical misinformation’s psychological implications must be considered when different interventions regarding frontline healthcare workers during the COVID-19 pandemic are implemented.

## 1. Introduction

As the coronavirus has spread across the world, so too the misinformation about it was exploded. The first cases of COVID-19 in Romania emerged in March 2020. By October, there were 222,559 infected, 6681 deaths, and 27,280 people in isolation. As the virus spreads across the country, the need for information has become a daily preoccupation for many people. Romanian Government created a Strategic Communication Group, which is qualified to communicate information about COVID-19 cases, treatment, and other social and medical implications. Nevertheless, most people rely on social media and look for information on social media platforms instead of using official communication channels.

Before the outbreak of COVID-19, people in many countries already relied on social media to gather information and news. Since the outbreak at the end of 2019, people worldwide return to social media, online press, or television to obtain as much information as they can [1]. A previous study showed that social media played an essential role in the COVID-19 outbreak in most countries. This role is highlighted in three areas: accurate information about the novel coronavirus was published on social media all over the world; fake news and misinformation about the outbreak were published daily on the internet, and social media has played an important role in creating and disseminating fear and panic about the outbreak worldwide [2].

According to *The New York Times*, medical misinformation about the novel coronavirus has been spread by ideologues who do not believe in modern medicine and scientifically proven treatments, like vaccines, and by profiteers who found an opportunity to promote cures or other wellness products [3]. Furthermore, specialists talk about an infodemic of misinformation. Infodemic is a blend of ‘‘information’’ and ‘‘epidemic’’, that refers to disseminating information, both accurate and inaccurate, about an important subject, as a disease. Once the information is spread, it becomes challenging to learn essential information about the topic. The word infodemic was first used in 2003 and has seen renewed usage since the outbreak of the COVID-19 crisis [4].

According to the World Health Organization (WHO), the COVID-19 related infodemic is just as dangerous as the virus itself. Misinformation, disinformation, and rumors during a health emergency, false preventive measures, fake remedies, conspiracy theories, and other incorrect information may have consequences beyond public health [5]. Since the COVID-19 outbreak in Romania, false information is spreading faster than the virus itself. The Romanian government and medical public health experts repeatedly warned against the negative consequences of some of the most viral false medical information, such as: the virus does not exist, the pharmaceutical giants invented the pandemic, vitamin C treats coronavirus, 5G is the source of the virus, people are paid to declare that they are infected with the novel coronavirus, and other misinformation. Infodemics can hamper a significant public health response and create confusion and distrust among people [6]. A past study illustrated the potential of using social media to conduct ‘‘infodemiology’’ studies for public health. Influenza A virus subtype H1N1 (H1N1) pandemic-related tweets on Twitter were used to disseminate official information from credible sources to the public and a source of opinions and experiences. The researchers proved the correlation between the prevalence of misinformation, terminology use, fear, and panic spread publicly, and the correlation between case incidence and public preoccupation [7]. During a pandemic, healthcare professionals should cooperate with the mass media and help identify the inaccurate, and misleading headlines that agitate members of the public, cause fear, impinge on public communication, and diminish countermeasures for the outbreak [8]. In a pandemic, people’s emotional reactions are likely to be very complicated and extensive, such as extreme fear and uncertainty. Furthermore, anxiety and distorted perceptions of risk will lead to negative social behaviors [9].

To our knowledge, little evidence is available on the impact of false information during the outbreak of the novel coronavirus (SARS COV2) on the general population and healthcare workers. Misinformation and fake health news in social media may constitute a potential threat to public health. Patients are more likely to mistrust the medical information and disrespect the preventive measures and the medical experts’ policies. Furthermore, considering previous studies on misinformation’s impact on mental health, false news may negatively impact medical staff. During the COVID-19 outbreak, faced with unprecedented challenges, doctors and nurses must manage levels of stress and trauma similar to ones usually experienced in war zones [10]. We already know from previous studies that during the outbreak of COVID-19, healthcare workers screened positive for moderate to severe levels of depression, anxiety, and stress [11].

Considering these factors, the sample of frontline healthcare workers were split into two groups based on the self-evaluated criteria: if they were or were not affected by infodemia in their activity, we aimed to investigate whether frontline healthcare workers who declared to be affected by false news show different levels of stress, anxiety, depression, and insomnia than frontline clinicians who do not consider themselves to be affected by infodemia related to the COVID-19 pandemic.

In summary, we hypothesize that frontline workers who were declared to be affected by false news are more likely to experience stress, anxiety, depression, and insomnia than healthcare workers who were not affected by fake news in their professional activity.

**Hypothesis 1** **(H1).** *Frontline healthcare workers who are declared to be affected by infodemia have a higher stress level than frontline clinicians who do not claim to be affected by false news.*

**Hypothesis 2** **(H2).** *Frontline medical clinicians affected by infodemia presented a higher level of anxiety than healthcare workers who are not affected by false information.*

**Hypothesis 3** **(H3).** *Frontline medical professionals who are declared to be affected by infodemia experienced a higher level of depression than their colleagues who are not affected by misinformation.*

**Hypothesis 4** **(H4).** *Frontline healthcare workers who claim to be affected by infodemia have a higher incidence of insomnia than frontline professionals who are not affected by false news.*

## 2. Procedure and Participants

We have surveyed frontline healthcare workers, emergency doctors, ICU doctors, and medical nurses from two Hospital Departments (Emergency and ICU) in Romania, namely the County Emergency Clinical Hospital Pius Brinzeu, Timisoara. The present study is a cross-sectional one; all data were collected from March to April 2020. The study was conducted following the Declaration of Helsinki and approved by the Ethics Committee of the County Emergency Clinical Hospital, No. 170/05.08.2019, as part of ongoing research considering the burnout syndrome and psychological implications healthcare profession. All gathered information was confidential; the participation was entirely voluntary, and written informed consent was obtained from all the participants.

The inclusion criteria concerned the categories of personnel who directly contact patients during the COVID-19 outbreak through the performed medical act, respectively, primary doctors, specialists, residents (trainees), ICU, and emergency medicine nurses. All data were collected online via a link sent by email. A total of 126 health professionals took part in the survey: 32 nurses and 94 physicians were questioned. Sociodemographic data were collected on gender (male or female), marital status (single, married, divorced, widowed), parental status (children; yes or no), profession (physician or nurse), technical title (trainee, specialist, primary or other), and specialty (ICU or emergency medicine specialist).

The Plan of measures for hospitals’ preparation in the COVID-19 pandemic, stated by the Romanian Ministry of Health Order number 533/03.29.2020, disposed of by the end of March 2020, that The County Emergency Hospital Pius Brinzeu Timisoara, Romania will take over the critical cases of patients infected with the novel coronavirus. The usual hospital activity was decreased by 80% regarding chronic cases to increase the hospital’s resources in treating COVID-19 patients [12]. By May 2020, 19,133 COVID-19 patients in Romania, 98,403 people in isolation, and 2993 people in official quarantine. Although Timis County had, by the end of May, 505 confirmed cases since the outbreak of the novel coronavirus crisis in Romania in early March, the increase in demand and changes to supply, redeployment of staff, extended work tasks the reorganization of hospital facilities, increase in donning and doffing personal protective equipment (PPE) and implementing new guidelines and protocols, caused tremendous psychological pressure for the frontline healthcare workers [13].

## 3. Materials and Methods

The Depression, Anxiety, and Stress Scale (DASS 21) is a reliable and suitable questionnaire to assess symptoms of common mental health problems, such as depression, anxiety, and stress. This scale’s essential function is to evaluate the severity of the core symptoms of depression, anxiety, and stress; thus, it supports our research questions. The DASS 21, as a self-report questionnaire consisting of 21 items, has 7 items per subscale: depression (e.g., “I couldn’t seem to experience any positive feeling at all.”—the Cronbach’s alpha for this scale was α = 0.88), anxiety (e.g., “I was worried about situations in which I might panic and make a fool of myself.”—the Cronbach’s alpha for this scale was α = 0.88), and stress (e.g., “I felt that I was rather touchy.”—the Cronbach’s alpha for this scale was α = 0.91). Depression refers to depressed mood, dysphoria, loss of interest and pleasure, anhedonia, and increased fatigue; anxiety refers to agitation, impatience, trouble concentrating, irritability, restlessness, difficulty relaxing, and difficulty falling asleep, while the third factor labeled stress refers to emotional or physical tension. The items are evaluated on a Likert scale, from 0 (did not apply to me at all) to 3 (applied to me very much) [14].

The Insomnia Severity Index (ISI) is a valid a reliable instrument to assess cases of insomnia in the population, with a good psychometric properties The ISI is composed of seven items (e.g., “How worried/distressed are you about your current sleep problem?”), rated on a five-point Likert scale (‘0’—not at all, ‘4’—extremely), and the time interval is ‘in the last two weeks’. Cronbach’s alpha for this scale was 0.91 [15].

Before the study’s beginning, we organized a focus group with respondents from the ICU and Emergency Department, doctors, and nurses. We disseminated two directions and created custom-made questions following these recommendations. The frontline healthcare workers suffered from the false news impact in two ways: they are exposed to medical misinformation and need to make an effort to discern the actual news, and on the other hand, the doctor-patient relationship is affected, patients been themselves exposed to false news and having troubles trusting the medical system and the healthcare specialists. Therefore, specific questions related to fake news influence on the frontline medical staff were created, such as: “Are you affected by fake news in the course of your professional activity?”, “In what way fake news affects you?”, “What is the word that best describes the media position (print, audiovisual, online press) regarding medical staff during the outbreak of COVID-19?”.

We have used the Statistical Package for Social Science (SPSS) v. 21 program (IBM Corp., Armonk, NY, USA). to test our hypothesis. The significance level adopted was *p* ≤ 0.05. Independent Student *t*-tests between two independent groups for all the variables were calculated.

## 4. Results

Demographic data were self-reported by the participants, as follows: gender (35.7% male and 64.3% female), marital status (42.8% single, 52.3% married, and 4.7% divorced), children (55.5% yes, 44.4% no), profession (74.6% physician, 25.3% nurse), staff category-doctors (45.2% trainee, 15% specialist, 16.6% primary, 23% other), and specialty (36.5% ICU and 63.4% EM) (Table 1), During the study period, the County Emergency Clinical Hospital Pius Brînzeu, Timisoara, was actively involved in the care of COVID-19 patients.

We split the sample of frontline healthcare workers into two groups based on the *criteria*: if they were or were not affected by fake news in their professional activity. We compared these groups concerning stress, depression, anxiety, and also insomnia (Table 2).

The frontline medical workers who were declared to be affected by false news (N_1_ = 43) were significantly more stressed (*t* = 3.04, *p* < 0.001) than healthcare workers who are not affected by misinformation related to pandemic time (N_2_ = 83), and this result offers support for Hypothesis 1. The healthcare workers who are affected by infodemia (N_1_ = 43) feel more anxiety (*t* = 1.91, *p* < 0.05) than healthcare workers who are not affected by false news (N_2_ = 83), supporting Hypothesis 2. Regarding Hypothesis 3, we found no difference in the level of depression between the frontline clinicians who are declared to be affected by false news (N_1_ = 43) and their colleagues who claim not to be affected by infodemia related to pandemic times (*t* = 1.54, *p* < 0.12). Consistent with Hypothesis 4, the frontline workers who are affected by misinformation suffer more from insomnia (*t* = 1.89, *p* < 0.05) than healthcare workers who are not affected by the infodemia related to pandemic time (N_2_ = 83).

Regarding the specific questions related to the false news impact on the frontline medical staff, we obtained the following results: 34% of frontline healthcare workers answered yes to the question: “Are you affected by false news in the course of your professional activity?”.

The most common answers to the question: “In what way fake news affects you?” were: “The doctor-patient relationship is affected. People distrust doctors and the medical system because they are misled by fake news.” (23% of the respondents), “It affects me emotionally.” (30% of the participants), and “It creates confusion.” (19% of the respondents).

The top three words found in the answers of the frontline healthcare workers regarding the question: “What is the word that best describes the media position (print, audiovisual, online press) regarding medical staff during the outbreak of COVID-19?” are: “appreciation”, (33% of the respondents), “distorted” (33% of the participants), and “objectivity” (15% of the respondents).

## 5. Discussion

The purpose of this research was to study if doctors and nurses who declared to be affected by false news show different types of psychological consequences than healthcare workers who do not consider themselves to be affected by fake news related to the COVID-19 pandemic.

The results were concordant with our predictions. Firstly, we found that almost half of the participants were affected by false news in their professional activity. The general population has been overwhelmed with information about COVID-19, including incorrect information and false information. Medical misinformation has centered around key themes: food and beverages as “cures,” hygiene practices, and medicines. Healthcare workers must take action by refuting or rebutting misleading health information and providing appropriate information [16].

As false medical news about the novel coronavirus spread across the world, healthcare workers found themselves in another battle, the second pandemic, an infodemic. As one study shows, the job of healthcare workers has changed. Academics need to publicly denounce wrongdoers and hold them accountable with scientific evidence in the battle with fake news during the outbreak of COVID-19 [17].

The COVID-19 pandemic is putting health systems and healthcare workers around the world under immense pressure. Besides treating patients with COVID-19, medical specialists need to battle with another enemy, fake news. The frontline medical workers who declared to be affected by false news believe that misinformation affects them in many ways, such as: “I am emotionally affected by fake news.”, “The doctor-patient relationship is affected by false medical news; patients distrust their doctors.” “It consumes time and energy to battle misinformation. It creates confusion. “People are scared, and it takes more time and energy from our part to calm them and explain scientific information.”, “Communication with patients influenced by fake news is difficult.”, “It affects our professional reputation and credibility.”, “It affects the general population’s trust in the medical system and doctors. People who suffer from time-sensible health problems are afraid to go to hospitals to get treatment. That makes our job harder. It is sad and problematic for all of us, the healthcare workers.”

Secondly, the frontline doctors and nurses who were declared to be affected by false news were significantly more stressed than healthcare workers who are not affected by medical misinformation related to the pandemic. Previous studies concerning the psychological sequelae observed during the SARS COV-1 in the 2003 outbreak revealed that healthcare workers experienced acute stress reactions [18]. In 2020, as one study shows, since the declaration of the coronavirus outbreak as a pandemic, some healthcare workers from different hospitals screened positive for moderate to extremely severe stress [19].

Work-associated stress affects healthcare workers, including doctors, nurses, auxiliary personnel, administrative staff, and other medical technicians. The three main work-related stress factors identified were: heavy workloads, the time-related pressure on the job, and extended working hours [20]. During a pandemic, frontline workers who are called upon to assist or treat those with COVID-19 may experience stress related to a physical strain of protective equipment, physical isolation, constant awareness, and vigilance regarding infection control procedures, pressures regarding procedures that must be followed [21]. Furthermore, as our study shows, frontline healthcare workers in Romania are influenced by false news and feel stress, among other psychological outcomes, in dealing with this particular factor concerning the public misinformation about the COVID-19 medical crisis.

Thirdly, the frontline medical workers who were declared to be affected by the infodemia felt more anxiety than healthcare workers who are not affected by false news related to pandemic time. The most frequently reported symptom during pandemic time is anxiety, both in the general population and medical staff. Many studies already demonstrated that frontline healthcare workers screened positive for moderate to severe anxiety during the outbreak of COVID-19. Before COVID-19, internet addiction was already recognized as a growing problem contributing to social anxiety, attention-deficit/hyperactivity disorder, and other wellness aspects, which may only intensify during a pandemic time [22].

Since the COVID-19 pandemic outbreak, the constant stream of information and fake medical news can be overwhelming for anyone, let alone clinicians already facing stressful challenges in their professional and personal lives [23]. False medical messages trigger feelings of fear and panic in public. When the message has an emotional impact, people are more inclined to share that information with family and friends. Despite our Government’s efforts to communicate efficiently and disseminate medical information to the public, false news continued to spread much faster than the virus itself, leaving the medical community on the frontline of another battle, with misinformation and disinformation regarding the coronavirus crisis.

Finally, the frontline healthcare workers who are declared to be affected by misinformation suffer more from insomnia than healthcare workers who are not affected by false news related to pandemic time. Previous studies on emergency medicine specialists in Romania demonstrated that work-related stress symptoms, such as sleep disorders, play an essential role in the medical staff’s mental health [24].

During a pandemic time, medical staff is placed under tremendous pressure, leading to many psychological reactions, including sleep disorders and low sleep quality, being present almost all the time [25,26]. A cross-sectional survey among healthcare workers treating patients with COVID-19 in China revealed that a significant proportion of participants experienced insomnia symptoms [27]. Having to face permanent dissemination of misinformation about the coronavirus, its treatments, evolution, impact, and even existence of the virus, the frontline medical workers felt the influence of this significant stress factor also, infodemia.

This research could be considered an initial attempt to integrate the false medical information stress-factor, among the other occupational stress causes during a pandemic time, which, to our understanding, is new and unique in Romanian frontline healthcare workers during the SARS COV-2 pandemic.

The results of this study should be evaluated, considering several limitations. One of the limits is the cross-sectional design. Our research cannot assess if there will be a change in variables over time. The relations found do not involve causal inferences between the studied variables. We attempted to compare the study groups concerning stress, depression, anxiety, and insomnia; further longitudinal research may contribute to a better understanding of ways in which the causality relationship regarding the false news effect on medical frontline healthcare workers and other psychological implications may occur. Another limit is the self-reported impact of the false news in the medical activity; the subjectivity of the cohort classification can be overcome in future research aiming at objective measurements of the false news factor. Despite the Romanian Strategic Communication Group’s effort to present and explain in real-time, when possible, the false medical information that appeared in the Romanian media, separating false information from actual news can seem daunting and may influence our study variables.

Moreover, the sample size was too small. Further research with a larger sample, such as a nation-wide study, should be performed to gain a complete image of the fake news influence on doctors and nurses during the pandemic time. Longitudinal studies could further strengthen our conclusions and evidence of the relationships between fake news and different psychological outcomes Further research is needed to test a regression model of the study variables and factors that may be associated with the exposure to fake news in the medical and general population. Future research also may improve our knowledge of the impacts of false medical news, from efficient tools to discern between true and false content to better develop our cognitive reflection and overcome other psychological implications due to the exposure to false news in the COVID-19 pandemic.

## 6. Conclusions

In conclusion, our findings suggest that frontline healthcare workers who are declared to be affected by false news show different levels of psychological manifestations such as anxiety, stress, and insomnia, during the outbreak of the COVID-19 pandemic. They should be considered in the aftermath of the COVID-19 crisis when policies and interventions for positive mental health and well-being among frontline medical staff are designed and implemented.

## Figures and Tables

**Table 1:** Demographic and professional characteristics of frontline healthcare workers.

Variables	Categories	Frequency	Percentage
Gender	Male	45	35.7
	Female	81	64.3
	Total	126	100
Marital status	Single	54	42.8
	Married	66	52.3
	Divorced	6	4.7
	Widower	0	0
	Total	126	100
Children	Yes	70	55.5
	No	56	44.4
	Total	126	100
Profession	Physician	94	74.6
	Nurse	32	25.3
	Total	126	100
Staff category-doctors	Trainee	57	45.2
	Specialist	19	15
	Primary	21	16.6
	Other	29	23
	Total	126	100
Specialty	ICU	46	36.5
	EM	80	63.4
	Total	126	100

**Table 2:** Statistical indicators of differences.

	Variables	N	Mean	*t*-Test	*p*
Stress	Affected by fake news	43	7.23	3.04	0.00
	Not affected by fake news	83	4.71		
Depression	Affected by fake news	43	4.79	1.54	0.12
	Not affected by fake news	83	3.59		
Anxiety	Affected by fake news	43	3.93	1.91	0.05
	Not affected by fake news	83	2.61		
Insomnia	Affected by fake news	43	10.86	1.88	0.05
	Not affected by fake news	83	8.66		

## Abbreviations

COVID 19	Coronavirus disease
WHO	World Health Organization
H1N1	Influenza A virus subtype H1N1
SARS COV2	Novel coronavirus
PPE	Personal protective equipment
The DASS-21	Depression, Anxiety and Stress Scale
SPSS	Statistical Package for Social Science
